# Supplementary material for: Folded network and structural transition in molten tin
Source: Nat Commun. 2022 Jan 10;13:126. doi: 10.1038/s41467-021-27742-2 (PMC8748439; doi:10.1038/s41467-021-27742-2)
Supplement: Supplementary file 1 — Supplementary Information [file 41467_2021_27742_MOESM1_ESM.pdf]

Supplementary Information

Folded Network and Structural Transition in Molten Tin

L. Xu et al.

Supplementary Note 1. Extraction of high-*q* structure factors of liquid Sn

Identifying subtle changes in the 3D liquid structures by means of 1D radial distribution function *g*(*r*), or its reciprocal-space counterpart, structure factor *S*(*q*), requires knowledge of *g*(*r*) or *S*(*q*) to a very high accuracy. In this work, the structure factors of *l*-Sn were derived from high-energy XRD and analyzed with an in-house computational code[1]. In what follows, we introduce the method employed in this work to extract the structure factors.

The diffraction intensity contains scattering signals from different sources, including background, scattering from the sample, and sample absorption effect:

$$I^{meas.}(q) = T(q) \cdot I^{samp}(q) + s \cdot I^{background}(q) \quad (1)$$

where $I^{meas.}(q)$ is the integrated diffraction signal from the image plate (see Supplementary Fig. 1a), and $I^{background}(q)$ is the diffraction intensity without the sample. $T(q)$ is the transmission coefficient as a function of momentum transfer *q*, which can be calculated according to textbook formulas for given sample geometry, thickness, and material. s is a scaling factor. $I^{samp}(q)$ includes sample coherent scattering, incoherent scattering, and an empirical correction:

$$\alpha \cdot I^{samp}(q) = I^{coh}(q) + I^{incoh}(q) + I^{empr}(q) \quad (2)$$

where α is the normalization factor to put the signal into atomic units, and I^{incoh} is the incoherent (Compton) scattering from the atoms in the sample which can be computed using the analytical formula provided by Balyuzi[2]. For the empirical correction (which accounts for other spurious effects such as multiple scattering, fluorescence radiation, temperature diffuse scattering, etc.), we adopted a modified function due to Thijsse[3]:

$$I^{empr}(q) = [\frac{a \cdot (0.75q / q_{max})^2}{1 + (0.75q / q_{max})^2} + b \cdot q + c \cdot q^2] I^{incoh} \quad (3)$$

where *a*, *b*, *c* are parameters to be optimized through the "correctness" of the structure factor *S*(*q*), which is related to $I^{\mathrm{samp}}(q)$ by:

$$S(q) = \frac{I^{coh}(q)}{f^2(q)} \quad (4)$$

where *f* is the atomic scattering factor for Sn, obtained from tabulated values.

The following three criteria were used to gauge the accuracy of $S(q)$

(1) The high-q rule:

At high-q values (e.g., $q > 12$ Å^{-1}), $S(q)$ should oscillate around 1. Therefore, we set

$$\mathrm{X}_1^2 = \frac{1}{ndata} \sum_{q_i = q_{mid}}^{q_{max}} [I^{coh}(q_i) - f(q_i)]^2 \tag{5}$$

(2) The sum rule:

According to Krogh-Moe[4] and Norman[5], the structure factor and the number density ρ_0 have the following relation:

$$-2\pi^2 \rho_0 = \int_0^\infty [S(q) - 1] q^2 dq \tag{6}$$

A second cost function can thus be formulated to serve as an additional constraint:

$$\mathrm{X}_2^2 = 2\pi^2 \rho_0 + \sum_{q_{min}}^{q_{max}} [S(q_i) - 1] q_i^{\,2} \delta q \tag{7}$$

Here, if the number density ρ_0 is not known *a priori*, it can be optimized together with other parameters. In the present study, it was stipulated from our *ab initio* MD simulation.

(3) The Kaplow rule

Kaplow *et al.*[6] observed that for small r, the reduced radial distribution function $G(r)$ has

$$G(r) = -4\pi r \rho_0 \tag{8}$$

Therefore, a third cost function was constructed to refine the optimization:

$$\mathrm{X}_3^2 = \frac{1}{ndata} \sum_{r_i = 0}^{\sigma} [G(r_i) + 4\pi r_i \rho_0]^2 \tag{9}$$

where $\sigma = 2$ Å and $G(r) = \frac{2}{\pi} \int_0^\infty q[S(q) - 1] \sin(qr) dq$.

Lumping the three criteria together, we defined a total cost function:

$$\chi^2 = \sum_{i=1}^{3} w_i \chi_i^2 \tag{10}$$

where w_i are fitting weights. The parameters *a, b, c, s,* and ρ_0 were optimized by minimizing χ^2 using the simulated annealing method[7] in this work.

Based on this approach, we were able to obtain accurate high-resolution pair correlation functions of *l*-Sn, enabling us to probe into subtle structural changes, e.g., the peak-splitting of $g(r)$ in the intermediate range around 6.0 Å. The optimized structure factors are shown in Supplementary Fig. 2 in comparison with previous neutron scattering data at selected temperatures. Our XRD data and previous neutron scattering data agree with each other, except for small discrepancies at the small-q values. The experimental radial distribution functions also agree well with *ab initio* MD results in terms of peak position and shape, showing the predictive capability of *ab initio* modeling.

Supplementary Note 2. Obtaining isothermal compressibility of liquid Sn from small-angle X-ray scattering experiment

The data processing of SAXS was similar to Supplementary Note 1. Our first step was to remove the background following Supplementary Eq. 1. In this case, the transmission coefficient $T(q)$ was explicitly determined by determining the ratio of the beam flux of the X-ray beam before and after the sample was inserted. Hence, Supplementary Eq. 1 is simplified as:

$$I^{sample}(q) = I_s^{meas.}(q) - I_s^{background}(q) \tag{11}$$

where $I_s^{meas.}(q)$ and $I_s^{background}(q)$ are the scaled scattering intensity with and without the sample in the capilary. The integrated SAXS intensities of the sample and background are shown in Supplementary Fig. 8a, respectively. Note that the humps on the diffraction profiles (for both sample and background) arose from the scattering of Kapton tape in our experiment, which was systematically removed (Supplementary Eq. 11) in subsequent data processing. The SAXS data at very small angles (q < 0.18 Å^{-1}) were unreliable due to interferences of optical devices, which were discarded in our derivation of $S(q)$. The sample intensity was converted to the structure factor following Supplementary Eq. 4. Due to lack of absolute calibration in SAXS experiments, Supplementary Eq. 4 was expressed as:

$$\alpha \cdot S(q) = \frac{I^{sample}(q)}{f^2(q)} \quad (12)$$

where the atomic form factor $f(q)$ was obtained from tabulated values, and α is a normalization factor to bring the $S(0)$ value at 530 K to match the experimental isothermal compressibility data available in the literature[8].

From thermodynamic fluctuation theory[9,10], the isothermal compressibility as a function of temperature is linked to the structure factor $S(0)$ (i.e., density fluctuation at the long-wavelength limit) by the relation:

$$S(0) = nk_B T\kappa_T \quad (13)$$

where k_B is the Boltzmann constant, T is the absolute temperature, and n is the atomic number density[11]. $S(0)$ was obtained by extrapolating the structure factor in the range of $0.18 < q < 0.4$ Å^{-1} to $q = 0$ using a quartic polynomial, as shown in Supplementary Fig. 8b.

Supplementary Note 3. Isothermal compressibility of liquid Sn derived from *ab initio* MD

Based on thermodynamic fluctuation theory[9,12], the mean-square fluctuation in number density ρ ($\rho = N/V$) at a constant temperature T is given by:

$$\left\langle (\delta\rho)^2 \right\rangle = \left(\frac{k_B T\rho}{V}\right)\left(\frac{\partial\rho}{\partial P}\right)_T \equiv \frac{k_B T\rho^2}{V}\kappa_T \quad (14)$$

where κ_T is isothermal compressibility, k_B is Boltzmann constant, T is temperature, and V is volume. The above equation can be expressed in terms of volume fluctuation:

$$\kappa_T = \frac{\left\langle (\Delta V)^2 \right\rangle}{k_B T V} \quad (15)$$

To compute κ_T of *l*-Sn, our goal was to obtain the mean-square fluctuation of volume, $\left\langle (\Delta V)^2 \right\rangle$.

To this end, a large ensemble consisting of 288 atoms (e.g., V = 31.4 Å^3 at 600 K) was subjected to NPT *ab initio* MD simulations at desired temperatures. At each temperature, the volume was controlled by the method of Parrinello and Rahman[13,14] combined with a Langevin thermostat as implemented in VASP. It was found that the mean-square volume fluctuation follows a normal distribution and the result converges within approximately 200,000 timesteps (the data are not shown), from which the isothermal compressibility was computed and plotted in Fig. 2d. The error bars in Fig. 2d correspond to the standard deviations of volume fluctuations.

To cross-check the accuracy of the isothermal compressibility computed with the above method, we further computed κ_T from the following relation:

$$\kappa_T = -\frac{1}{V}\left(\frac{\partial V}{\partial P}\right)_T \tag{16}$$

In doing so, we established how the ensemble pressure P changes with uniform dilation of *l*-Sn. At each temperature, by varying the volume from -5% to 5%, a series of AIMD simulations were carried out in NVT ensembles to obtain the statistical mean of the pressure at each volume. The isothermal compressibility was thus calculated by fitting the pressure and volume relationship employing a second-order Brich-Murnaghan equation-of-state[15]. The as-obtained isothermal compressibility data at selected temperatures were juxtaposed with the data obtained from the fluctuation theory in Fig. 2d, showing satisfactory agreement between the two methods.

One should note that the isothermal compressibility values obtained with AIMD are consistently larger than the experimentally reported ones. This discrepancy concerns primarily with the accuracy of the DFT treatment in dealing with physical properties in general. For instance, with the current DFT treatment (*e.g.*, projector augmented-wave treatment and generalized-gradient approximation)[13,14], the theoretically calculated bulk modulus of α-Sn at 300 K (~33 GPa, this work) is roughly 62 % of the experimental value (53 GPa, ref. [16]), which justifies the differences observed between the experimental and theoretical isothermal compressibility.

Supplementary Note 4. Liquid excess entropy obtained with a multiple-histogram method (MH)

The multiple histogram method (MH) is a technique originally proposed by Ferrenberg and Swendsen[17-19] to obtain free energies and entropies from a discrete sampling of energy.

In a canonical ensemble, the distribution of potential energy E at a given temperature is given by

$$P(E,T) = \frac{\Omega_c(E)e^{-\beta E}}{Z_c(T)} \tag{17}$$

where Ω_c is the configurational density of states; β is inverse temperature $1/k_B T$; $Z_c(T)$ is the configurational integral (i.e., the configurational part of the partition function):

$$Z_c(T) = \int \Omega_c(E)e^{-\beta E} dE \tag{18}$$

The configurational density of states is related to the dimensionless configurational entropy as

$$S_c(E) = \ln \Omega_c(E) \tag{19}$$

From the energy distribution $P(E,T)$ at a given temperature sampled in a canonical ensemble, we might extract the configurational entropy S_c by inverting the above equations:

$$S_c(E) = \ln P(E,T) + \beta E + \ln Z_c(T) \tag{20}$$

This equation can also be rewritten as:

$$S_c(E) = \ln P(E,T) + \beta E - \beta A_c(T) \tag{21}$$

where $A_c(T) = -k_B T \ln Z_c(T)$ is the configurational part of the Helmholtz free energy $A(T)$, which has the following expression:

$$A(T) = A_c(T) + Nk_B Tln[N!\Lambda(T)^{3N}] \tag{22}$$

where $\Lambda(T)$ is the de Broglie thermal wavelength[20].

We list two equations based on which our histogram-based code was developed to derive the configurational entropy:

$$\ln P(E,T) = S_c(E) - \beta E + \beta A_c(T) \tag{23}$$

$$A_c(T) = -k_B T \ln \int e^{S_c(E)-\beta E} dE \tag{24}$$

The Ehrenfest definition of second-order phase transition requires that the second derivative of the free energy be discontinuous at the transition temperature. Therefore, in our analysis, we sought to derive a twice-differentiable free energy curve with respect to energy or temperature (strictly speaking, in the vicinity of the transition region). To achieve this, we expressed the configurational entropy with cubic spline interpolation[7]:

$$S_c(E) = c_0 + c_1 E + \sum_{k=2}^{n} c_k (E - E_k)^3 \tag{25}$$

where c_k are spline parameters to be determined and E_k are spline knots evenly distributed between two predefined energy bounds. In our spline representation of $S_c(E)$, 15 knots were used (i.e., n = 16). Given the analytical form of the configurational entropy, the integral form of the free energy can be numerically assessed according to Supplementary Eq. 24. In this approach, the entropy can

only be optimized within an additive constant (a shift of S_c will be counterbalanced by the change of the integrated free-energy term following Supplementary Eq. 23), therefore, c_0 is arbitrarily fixed *a priori*.

To optimize the spline parameters c_k, we defined a cost function by comparing the theoretical probability $\ln[P(E,T)]\Delta E\,|_{E=E_i,T=T_j}$ (see Supplementary Eq. 23. ΔE is the bin size of the histograms) with the actual probability $\ln[P(E_i,T_j)]$ from the energy histograms sampled at a specified simulation temperature T_j (e.g., T_j = 600 K) :

$$\begin{aligned}&\ln[P(E,T)]\Delta E\,|_{E=E_i,T=T_j} - \ln[P(E_i,T_j)] \\ &\qquad \equiv [\mathrm{S}_c(E_i) - \beta_j E_i + \beta_j A_c(T_j)]\Delta E - \ln[P(E_i,T_j)]\end{aligned} \tag{26}$$

In practice, in order to improve the sampling statistics (the tails of the histograms are poorly sampled), we adopted a histogram reweighting technique, and the final cost function is redefined as:

$$\mathrm{X}^2 = \sum_{j=1}^{M}\sum_{i=1}^{N} P(E_i,T_j)\{[\mathrm{S}_c(E_i) - \beta_j E_i + \beta_j A_c(T_j)]\Delta E - \ln[P(E_i,T_j)]\}^2 \tag{27}$$

where M refers to the number of independent temperatures at which *NVT* simulations were conducted, and N is the number of histogram bins. Supplementary Fig. 9 illustrates the histograms sampled at selected temperatures T_j. We adopted the simulated annealing technique[7] to optimize the cost function.

To achieve thermodynamically convergent results, 18 temperatures were chosen to ensure adequate overlaps in the potential energy distributions. Satisfactory statistics on energy distribution were achieved by long-time *ab initio* MD simulation, *ca*. 300,000 time-steps at each temperature. MD simulations were performed in canonical ensembles (288 atoms) using the Nose-Hoover thermostat for temperature control[21]. The total number of state points for the thermodynamics analysis was tallied to 5×10^6.

Having obtained the configurational entropy $S_c(E)$, its temperature dependence could be derived from $\frac{1}{T} = \frac{\partial S_c(E)}{\partial E}$ (Supplementary Fig. 10). Note that the canonical configurational entropy $S_c(E)$ differs from the excess entropy of liquid S_{ex} (defined as the liquid entropy in excess of that

of the ideal gas) by an additive constant: $S_c = S_{ex} + S_c^{ideal}$, where S_c^{ideal} is the configurational entropy of the ideal gas at a given volume and $S_c^{ideal} = k_B \ln \frac{V^N}{N!}$=const.

Supplementary Note 5. Atomic relaxation dynamics in liquid Sn

The self-intermediate scattering function $F_s(q,t)$ characterizes the relaxation time of *l*-Sn. The $F_s(q,t)$ is defined as the Fourier transform of the van Hove function[22]. Instead of Fourier transformation, these functions can also be directly computed from the atomic trajectories[22]:

$$F_S(q,t) = \frac{1}{N} \sum_{j=1}^{N} \left\langle \exp[iq \cdot (r_j(0) - r_j(t))] \right\rangle \tag{28}$$

where q is the wave vector, and N is the number of particles. The relaxation time τ_α is defined as the time it takes for $F_s(q,t) = 1/e$ (see Fig. 3b in main text).

Supplementary Note 6. Chemical bonding analysis based on electron localization function

The interaction between the center atom and a coordination atom is considered a bond. The primary signature of a covalent bond is the sharing and localization of valence electrons between the bonding atoms. The degree of localization and the relative strength of atomic bonds can be assessed by the electron localization function[23,24],

$$ELF = \frac{1}{1 + (D_\sigma / D_\sigma^0)^2} \tag{29}$$

where D_σ is a measure of Pauli repulsion and scales with the probability density of finding another same-spin electron near the reference electron. D_σ^0 is the D_σ value in a homogeneous electron gas having the same local spin density. ELF = 0.5 represents the same level of Pauli repulsion as in the homogeneous electron gas, and a higher ELF value indicates that the electrons are more localized (e.g., ELF = 1.0 can be interpreted as perfect localization). We used VASP to quantify the ELF values[25,26] on a 100 × 100 × 100 grid in the cubic box. To characterize the bonding between any two atoms, we define the spatially averaged ELF value within an enclosed sphere Ω in the middle of two atoms:

$$\chi = \frac{1}{\Omega} \int_\Omega \frac{1}{1 + \left[D_\sigma(r) / D_\sigma^0 \right]} \mathrm{d}v \tag{30}$$

In this work, we set a radius of 0.35 Å for the enclosed sphere to obtain χ. Note that bond character value χ universally characterizes the bonding nature of the atoms regardless of the absolute electron charge density of the system in question. A similar method was previously adopted to define the chemical bonding in a chalcogenide glass[27].

Supplementary Note 7. CRN structure for LDA-Sn versus FN structure for *l*-Sn

Group IVA elements Si and Ge were demonstrated to have tetrahedrally coordinated low-density amorphous (LDA) structures[28,29], which can be described by the CRN model[30]. Knowledge of the structure of amorphous Sn, however, was not established. Since Sn belongs to the same group with s^2p^2 valance electrons, it is thus intriguing to inquire whether the CRN type low-density Sn exists. Currently, no experimental information is available on the structure of amorphous Sn, which can be synthesized in the laboratory, e.g., by vapor deposition onto very cold substrates[31,32].

Based on first-principles modeling, we are able to demonstrate the existence of metastable LDA-Sn at low temperatures. We first constructed an ideal tetrahedrally-coordinated CRN structure of Sn employing the WWW bond-switching algorithm[30]. The as-prepared amorphous Sn was fully relaxed with *ab initio* molecular mechanics at a constant pressure of zero. The relaxed LDA-Sn was found to be mechanically stable and to maintain a nearly perfect tetrahedral coordination; that is, the fully expended random tetrahedral network of Sn. To examine the thermal stability of the LDA-Sn, the as-relaxed CRN structure was heat treated by means of constant-pressure (at zero pressure) *ab initio* MD at a heating rate of 5×10^{12} K/s. Upon increasing temperature, the structure reaches its stability limit at 450 K, followed by a transition to high-density liquid (HDL) Sn accompanied by a volume collapse of ~20%. We now have two disordered structures of Sn, the LDA-Sn and the metallic HDL-Sn. Supplementary Fig. 19 displays the computed structure factor of LDA-Sn at 300K. Also compared are the tetrahedral order parameters for the CRN LDA-Sn and the folded-network structure of *l*-Sn at 450K (Supplementary Fig. 20).

Supplementary Note 8. Relaxation dynamics and lifetime of atomic bonds in liquid Sn

Atomic packing in *l*-Sn departs from the hard-sphere model and hence the Voronoi method[33] is no longer suitable for evaluating the coordination number (CN). Alternatively, the CN was directly obtained by counting the number of atoms within a cutoff distance. At the melting temperature of 580 K, Sn atoms are found to accommodate 8~9 atoms in the first atomic shell.

The lifetime of the atomic bonds was analyzed through the partial bond autocorrelation function, defined as:

$$\Psi_B^i(t) = \left\langle \Theta[N(i,t_0),t_0] \cdot \Theta[N(i,t_0),t_0+t] \right\rangle \tag{31}$$

where $N(i,t_0)$ is the atom number of the ith nearest neighbor at time t_0; $\Theta(N,t_0)$ indicates whether atom N is a coordination atom at time t_0. If yes, $\Theta(N,t_0)=1$, otherwise $\Theta(N,t_0)=0$. The angular bracket refers to the ensemble average. It can be seen that, for an arbitrary atom, if the ith nearest neighbor remains a coordination atom after time t, we say that the ith atomic bond persists within time t, that is, $\Theta\left[N(i,t_0),t_0\right]\cdot\Theta\left[N(i,t_0),t_0+t\right]=1$.

From the calculated $\Psi_B^i(t)$ shown in Supplementary Fig. 15a, the bond relaxation dynamics of *l*-Sn can be briefly summarized as follows:
(1) The bond relaxation time, τ_b, defined as $\Psi_B^i(\tau_b)=1/e$, is found to vary with bond lengths. The covalent bonds exhibit a slightly longer relaxation time than the metallic bonds. (2) During short times $t < \tau_b$ ($t < 1.0$ ps), the covalent bonds relax noticeably slower than the metallic bonds. (3) For extended times $t > \tau_b$, the relaxation dynamics of the long-lived metallic and covalent bonds converge, owing to the fluctuating bonding nature of the atomic bonds discussed below.

The lifetime distribution of the atomic bonds can be derived from $P_b^i(t) = -\frac{\partial \Psi_B^i(t)}{\partial t}$, which is plotted in Supplementary Fig. 15b as a probability profile. It can be seen that the distribution profiles for the covalent and metallic bonds show slight discrepancies in the short-time window but exhibit almost identical behavior with prolonged relaxation time. This is consistent with the observation that the chemical bonding has a bifurcated nature and fluctuates between covalent and metallic bonding, as confirmed by the cross bond-bond correlation function, defined as:

$$\Psi_B^c(t) = \left\langle \Theta^c\left[N(i,t_0),t_0\right] \cdot \Theta^m\left[N(i,t_0),t_0+t\right] \right\rangle \tag{32}$$

where superscript c denotes covalent bonding and superscript m denotes metallic bonding. Our analysis of $\Psi_B^c(t)$ confirms the mutual transition between covalent and metallic bonding of *l*-Sn. Interestingly, the bond relaxation dynamics of *l*-Sn resembles the bifurcated hydrogen bonding

observed in water. A schematic diagram is drawn to show the transition from covalent to metallic bonding in *l*-Sn as in Supplementary Fig. 16.

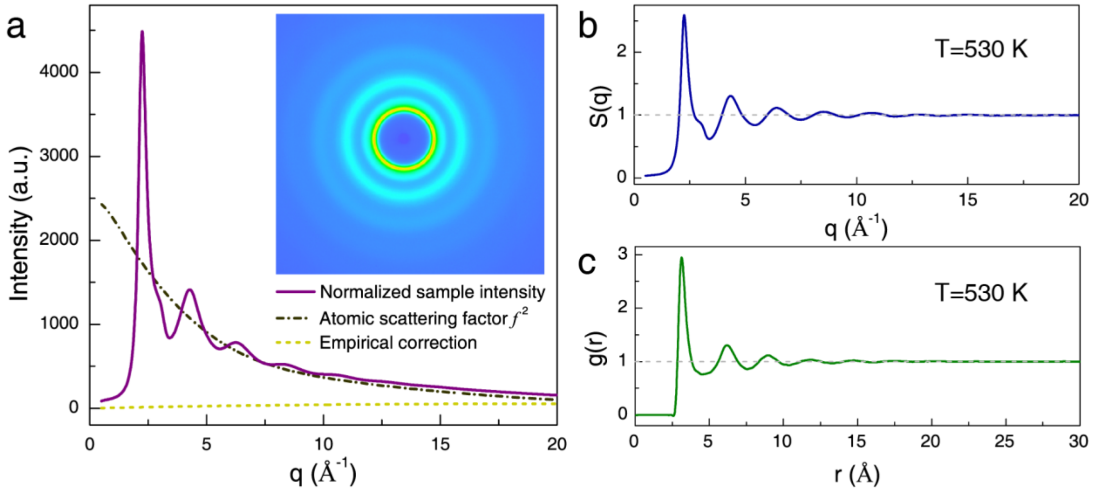

Supplementary Fig. 1. Extracting structure factor *S*(*q*) from high-energy XRD patterns. (a) Raw diffraction intensity integrated from a high-energy X-ray diffraction pattern of *l*-Sn. The inset shows a 2D image pattern recorded on the image plate. The normalized sample scattering intensity, atomic scattering factor, and empirical corrections are considered in structure factor optimization (see Supplementary Note 1). **(b)** Optimized structure factor of *l*-Sn at 530 K truncated at q_{max} = 20 Å^{-1}. **(c)** Radial distribution function obtained via Fourier transformation of the structure factor.

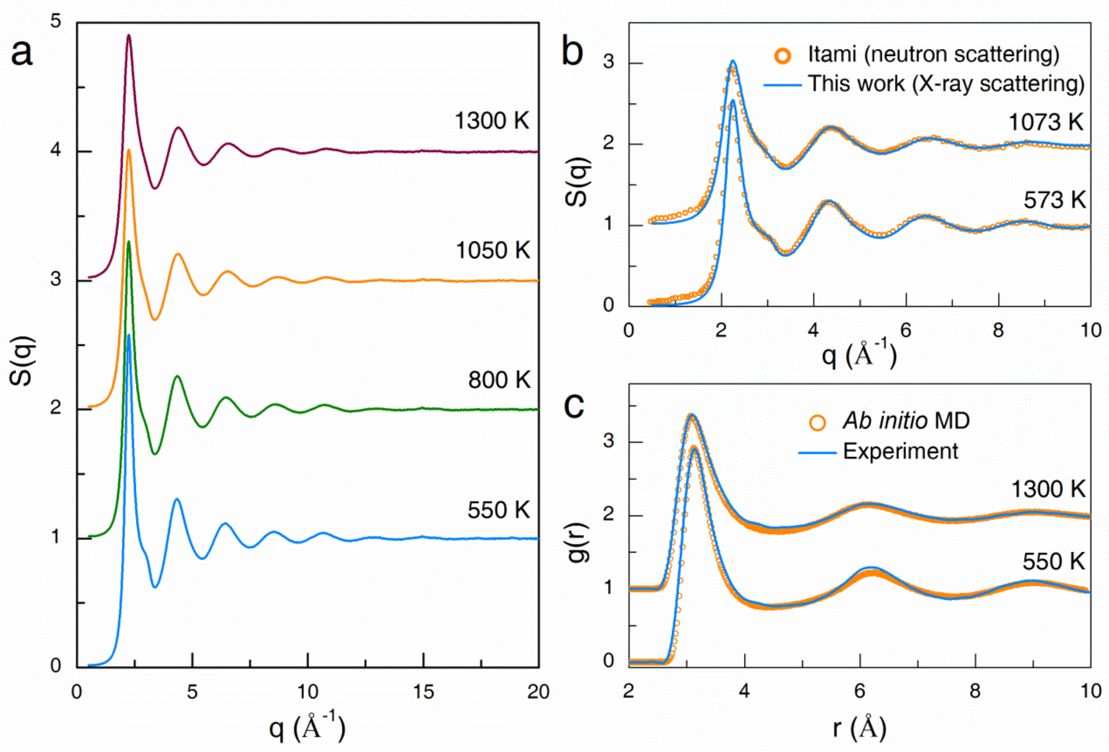

Supplementary Fig. 2. Structure factor $S(q)$ and radial distribution function $g(r)$ of liquid Sn obtained from experiment and AIMD. (a) Structure factors $S(q)$ of *l*-Sn at selected temperatures. **(b)** Comparisons of the structure factor $S(q)$ obtained from the present experiment (solid lines) and the previous neutron scattering experiment (open circles, ref. [34]). **(c)** Comparisons of the radial distribution function $g(r)$ between the present experiment (solid lines) and AIMD simulation (open circles). For the AIMD $g(r)$ data, the radial distance was scaled by a factor of 0.995.

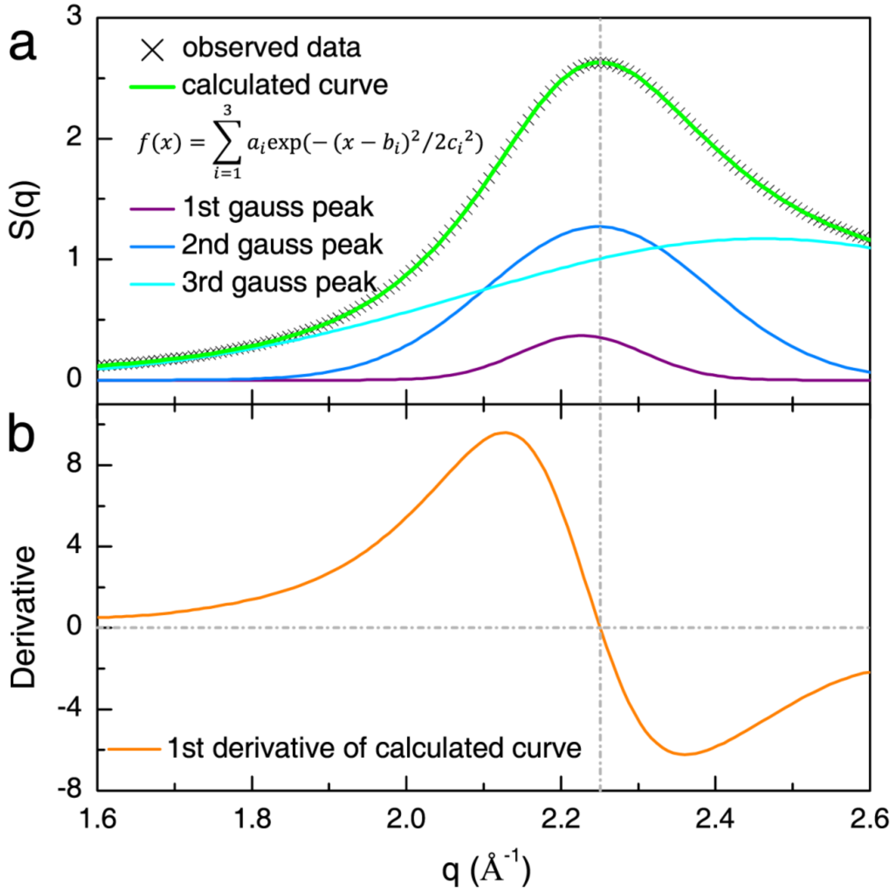

Supplementary Fig. 3. Mathematical treatment to find the peak position of *S*(*q*). (a) *S*(*q*) is fitted with multiple Gaussians. **(b)** The peak position corresponds to the *q* value where the first derivative of the fitted curve equals zero.

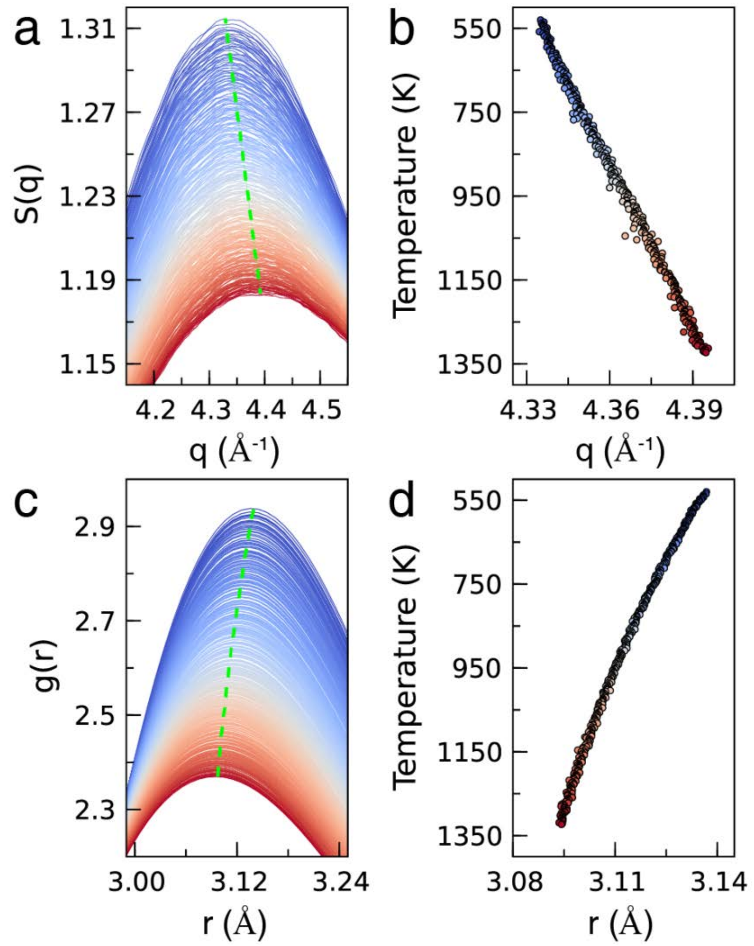

Supplementary Fig. 4. The evolution of *S*(*q*) and *g*(*r*) of liquid Sn. (a) and (b) Magnifications of the second peak of *S*(*q*) and temperature dependence of the peak position. **(c) and (d)** Magnifications of the first peak of *g*(*r*) and temperature dependence of the peak position. Monotonic peak shifts are found for the second peak of *S*(*q*) and the first peak of *g*(*r*) with increasing temperature.

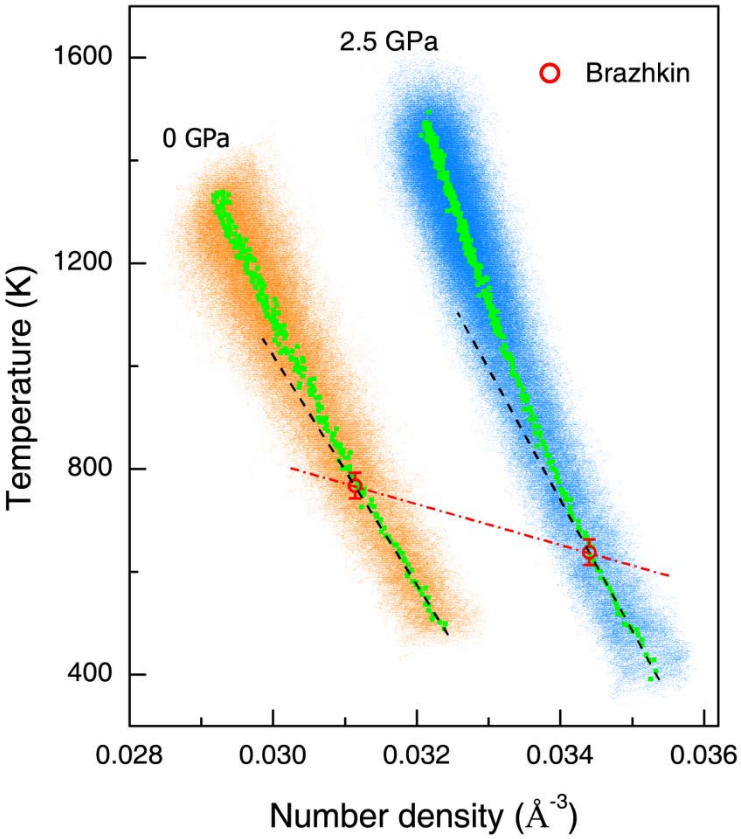

Supplementary Fig. 5. Number density of liquid Sn as a function of temperature at given pressures from AIMD. AIMD was conducted in NPH ensembles (N = 288 particles). The pressure was controlled by Langevin dynamics and the temperature was regulated by adding enthalpy to the system periodically. The orange and blue scatters are the raw data from AIMD at 0 and 2.5 GPa, respectively. The green symbols are the average value per 1000 points in the raw data showing that the number density changes with temperature. The number density data can be fitted piecewise linearly by $\rho = 0.0344 - 4.2336 \times 10^{-6} T$ (T < ~840 K) and $\rho = 0.0338 - 3.5193 \times 10^{-6} T$ (T > ~840 K) at 0 GPa, and $\rho = 0.0369 - 4.0351 \times 10^{-6} T$ (T < ~648 K) and $\rho = 0.0360 - 2.6463 \times 10^{-6} T$ (T > ~648 K) at 2.5 GPa. The fitted lines in the low-T regime are plotted to guide the eye. Brazhkin et al. has reported plausible phase transition temperatures at both pressures for *l*-Sn. The Brazhkin data (red open circle, refs. [35,36]) for the two pressures are also plotted for comparison. The inflection on the linear relationship of density vs. T suggests a structural transition in *l*-Sn, consistent with other experimental evidence discussed in the main text. The enthalpy changes as a function of temperature are plotted in Supplementary Fig. 6.

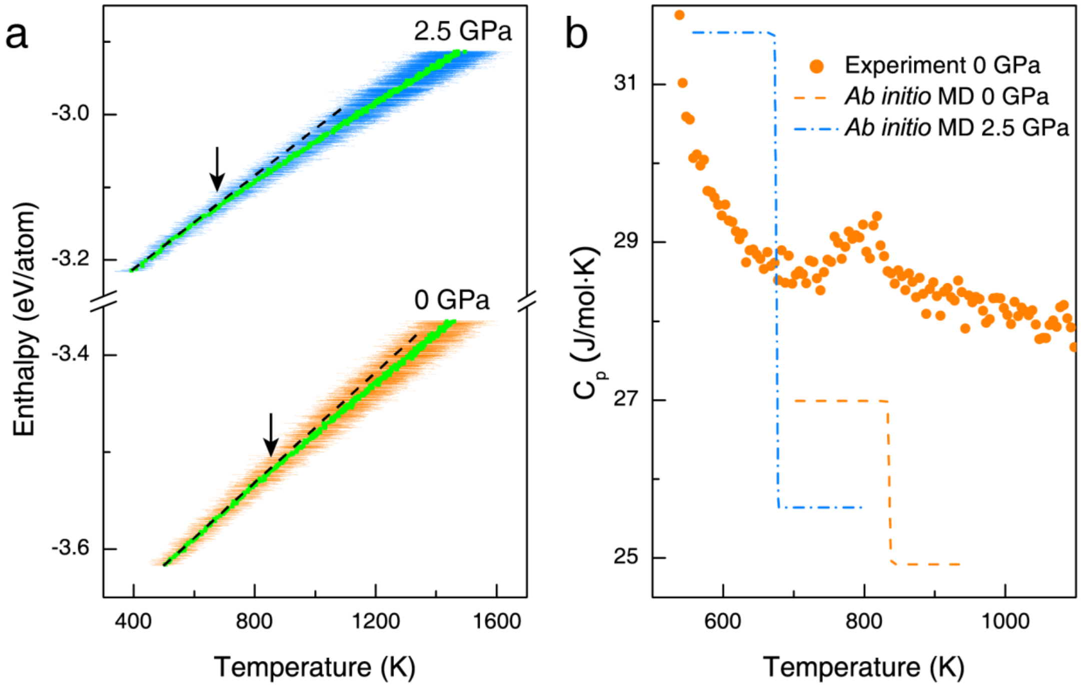

Supplementary Fig. 6. Enthalpy and C_P of liquid Sn as a function of temperature at given pressures obtained from NPH AIMD. (a) The enthalpies of *l*-Sn as a function of temperature at given pressures. The enthalpies can be fitted piecewise linearly by $H = -3.7570 + 2.7973\times10^{-4} T$ ($T <$ ~835 K) and $H = -3.7391 + 2.5830\times10^{-4} T$ ($T >$ ~835 K) at 0 GPa, and $H = -3.3462 + 3.2813\times10^{-4} T$ ($T <$ ~674 K) and $H = -3.3042 + 2.6578\times10^{-4} T$ ($T >$ ~674 K) at 2.5 GPa. **(b)** The heat capacity C_P as a function of temperature from experiment and AIMD. The AIMD C_P of *l*-Sn was derived from the slope of the enthalpy-temperature curves. A structural transition of *l*-Sn is inferred from the specific heat changes, which agree favorably with the experimental data. The density changes of the same simulations are given in Supplementary Fig. 5.

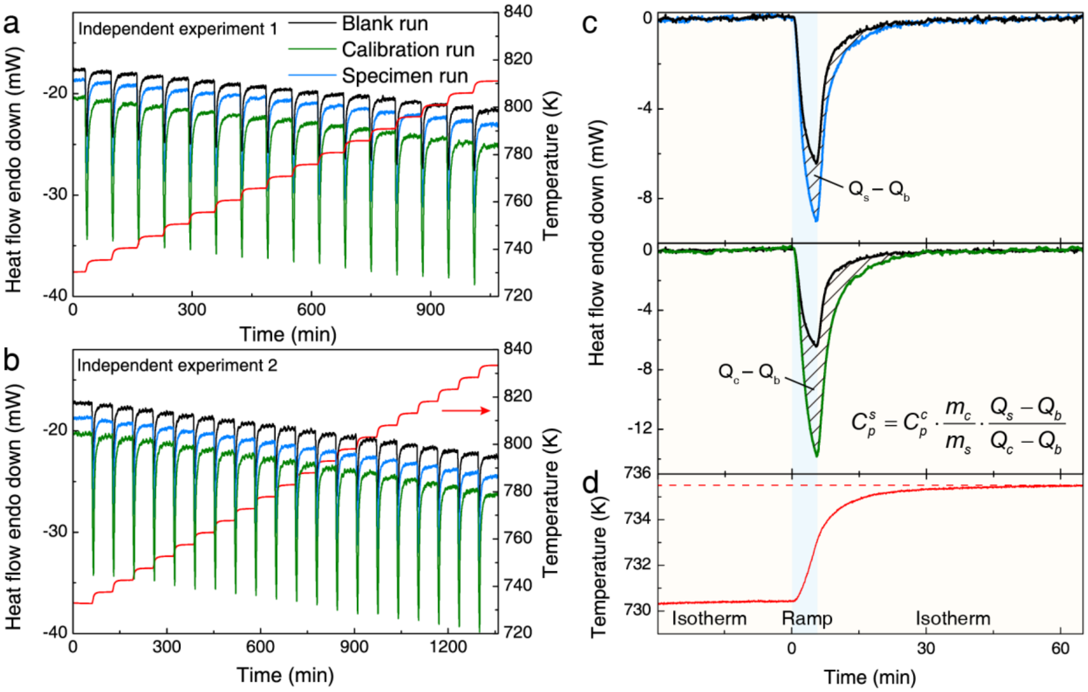

Supplementary Fig. 7. Accurate determination of heat capacity in the stepwise-scanning mode. (a) and (b) Two independent calorimetry experiments for the stepwise-scanning DSC data of *l*-Sn. (c) and (d) Mathematical treatment to obtain the heat capacity of *l*-Sn. From (d), one can see that the temperature during the thermal equilibration stage changes with time and gradually approaches a constant value in about 60 minutes. This justifies our use of 1 hour as the wait-time for thermal equilibration. In the inserted formula to calculate the heat capacity of the sample C_P^s, C_P^c is the known specific heat value of the calibrant (sapphire in this work); m_c and m_s are the masses of the calibrant and the sample, respectively. Q_s, Q_c and Q_b are the heat flows of the sample, calibrant and blank cell, respectively. The heat flows are integrated over the entire equilibration period, as seen in (c).

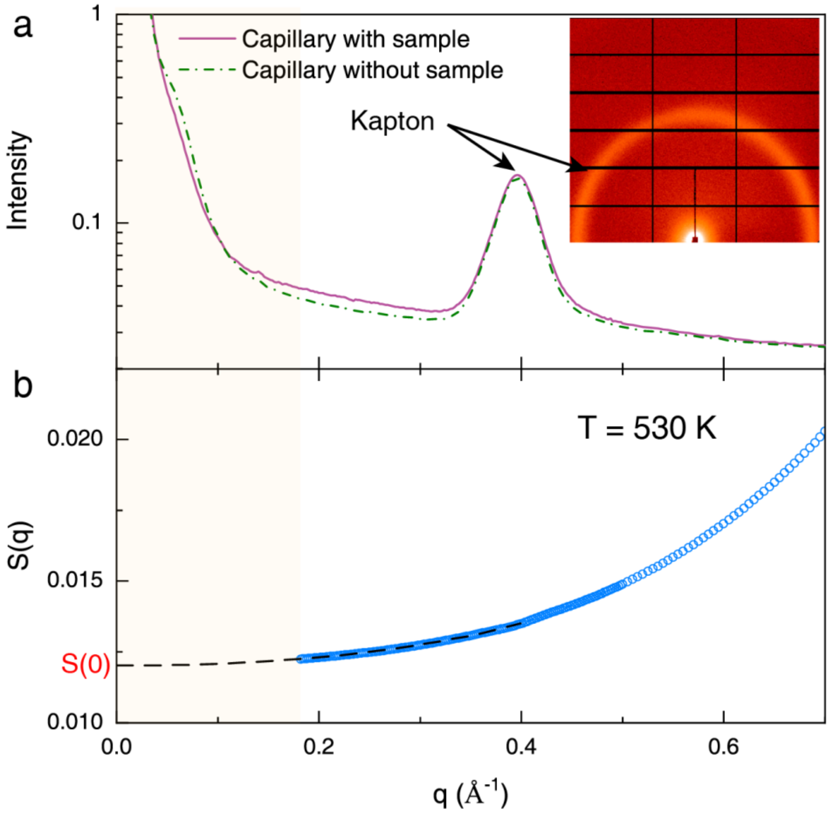

Supplementary Fig. 8. Extracting structure factor S(0) from SAXS patterns. (a) Small angle scattering intensities integrated from a SAXS pattern of *l*-Sn. The inset shows a 2D image pattern recorded on the image plate. The data processing of SAXS can be found in Supplementary Note 2. (b) Optimized structure factor of *l*-Sn at 530 K truncated at q_{min} = 0.18 Å^{-1}, which was extrapolated using a quartic polymomial to obtain $S(0)$.

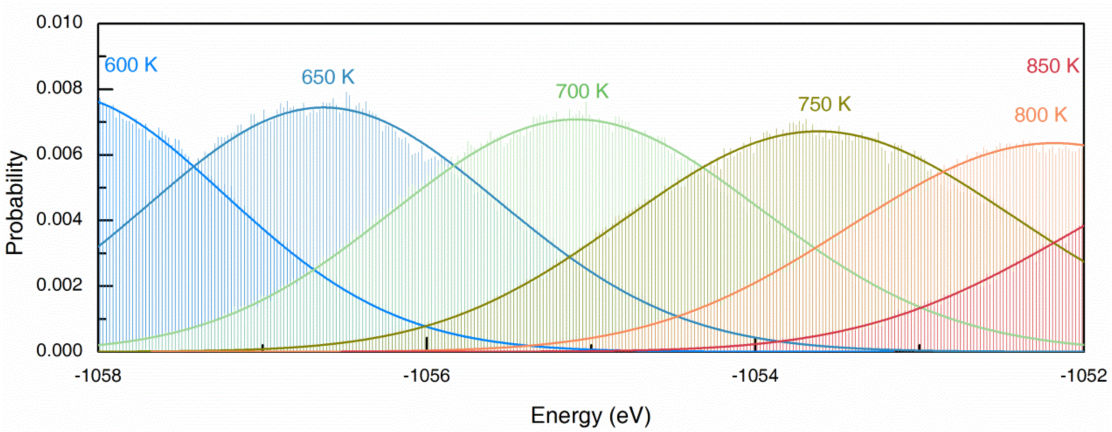

Supplementary Fig. 9. Histograms of potential energy sampled at different temperatures employing AIMD. Significant overlaps of the histograms are necessary for an accurate derivation of entropy and free energy. The lines correspond to theoretical probability distributions of potential energy based on the optimized configurational entropy from the multiple histogram method. AIMD simulations were conducted in NVT ensembles (N = 288 atoms, V = 30 Å^3/atom).

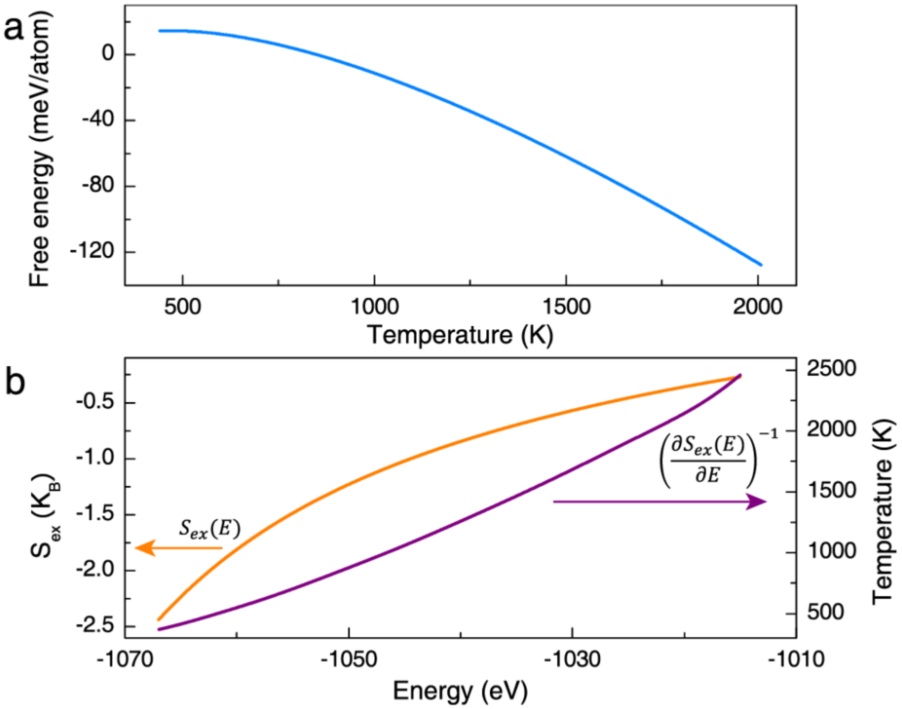

Supplementary Fig. 10. Derivation of thermodynamic properties of liquid Sn. (a) The Helmholtz free energy as a function of the temperature of *l*-Sn resulting from AIMD and the MH method. **(b)** Energy dependence of the configuration entropy and its temperature correspondence derived from $\frac{1}{T} = \frac{\partial S_{ex}(E)}{\partial E}$. The relationship between the excess entropy and the configurational entropy of *l*-Sn is explained in Supplementary Note 4.

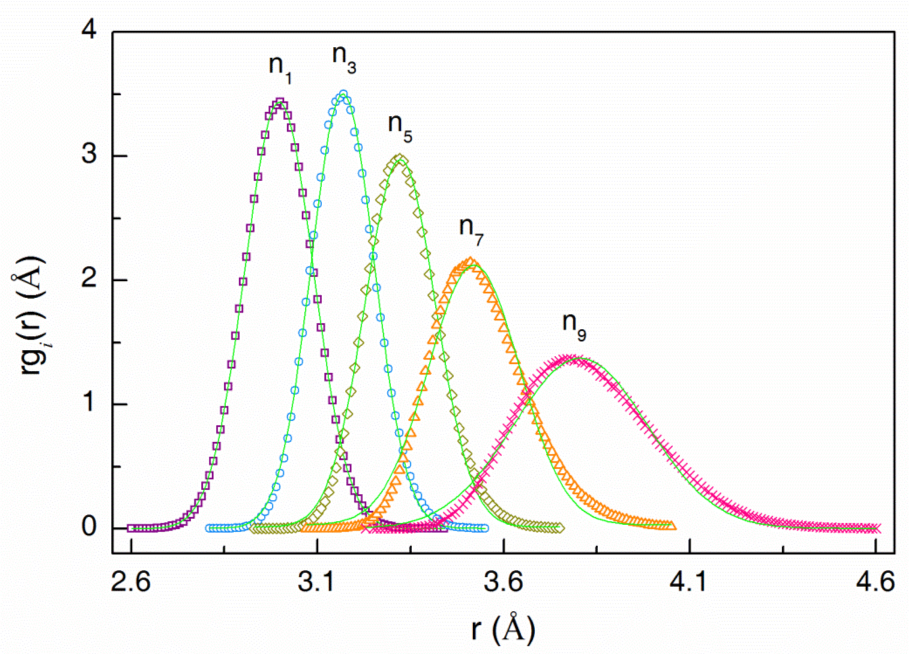

Supplementary Fig. 11. Radial distribution function $rg_i(r)$ of individual nearest neighbors in liquid Sn obtained from AIMD. The nearest-neighbor radial distribution functions $rg_i(r)$ can be satisfactorily described by Gaussian functions (*l*-Sn at T = 450 K, V = 30 Å^3/atom).

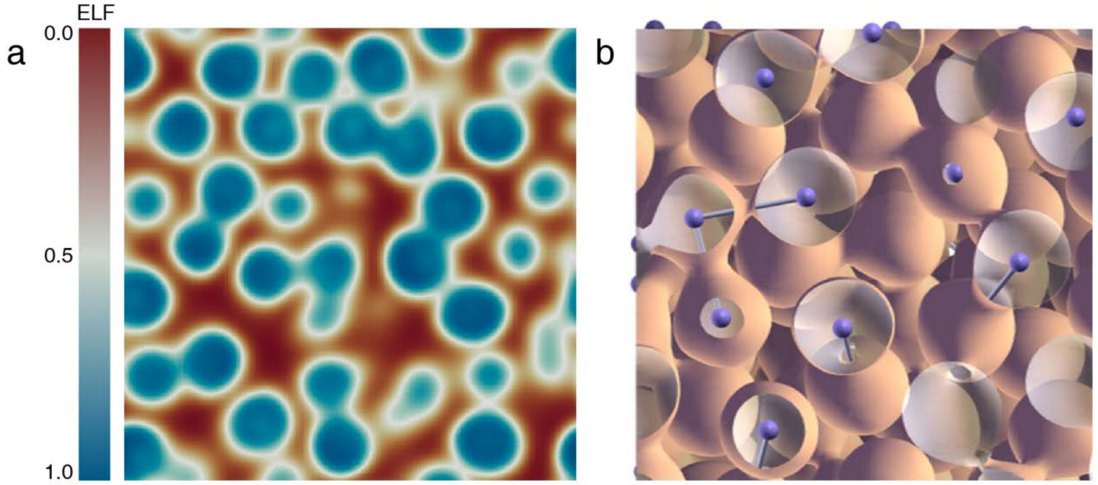

Supplementary Fig. 12. Projection of electron localization function (ELF) of liquid Sn. **(a)** Contour plot of ELF on a cut-plane of simulated *l*-Sn at 600 K. The bond character was numerically evaluated based on the ELF (see Supplementary Note 6). **(b)** A 3D isosurface of the ELF (*ELF* = 0.6) for *l*-Sn at 600 K, demonstrating covalent bonding of the atoms.

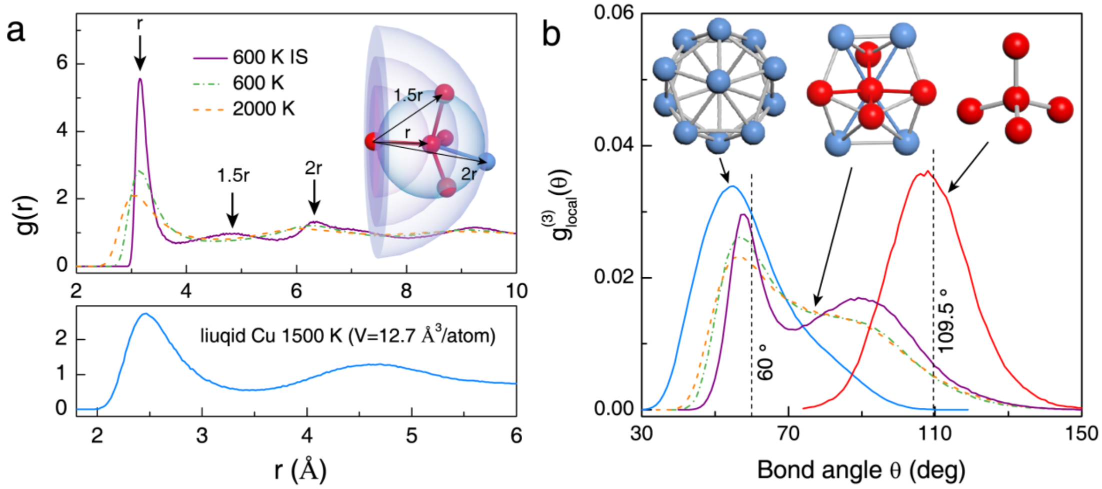

Supplementary Fig. 13. Structural characteristics of liquid Sn. (a) $g(r)$ of constant-density Sn liquids at various temperatures and the inherent structures corresponding to 600 K. Also shown for comparison is the $g(r)$ of liquid Cu (*l*-Cu) known to have HS-like atomic packing. The inherent structures of *l*-Sn correspond to local energy minima on the potential energy landscape, where forces on each atom vanish. Structural information can be readily revealed by analyzing the configurations of the inherent structures where the thermal effect is taken away. In this work, the inherent structures were obtained by conjugate-gradient energy minimization. The $g(r)$ of *l*-Sn exhibits distinctly different structural features than the HS liquid or the continuous random-network structure (see Supplementary Fig. 19). The positions of the first three peaks on the $g(r)$ of the inherent structures have characteristic ratios of 1:1.5:2. An illustration of the atomic arrangement is provided to explain the peak positions of the radial distribution function. The peak of $g(r)$ at position 2r indicates the presence of longer metallic bonds (blue bond and ball) opposing a covalent bond, in agreement with the folded-network model. **(b)** The local bond-angle distribution functions of *l*-Sn, the inherent structure of *l*-Sn, *l*-Cu and CRN-Sn, respectively. The local bond-angle distribution functions are labelled as follows: *l*-Sn (dashed lines, temperature as given in (a)) and the inherent structure of *l*-Sn (purple solid line), *l*-Cu (blue solid line) and CRN-Sn (red solid line). In calculating the local bond-angle distribution $g^{(3)}_{local}(\theta)$, we only consider the bonds that belong to the same tessellated tetrahedron in the first coordination shell (Note that the coordination polyhedron can be spatially tessellated into tetrahedra using the Voronoi method). As shown for the case of *l*-Cu where the atoms are closely packed, the $g^{(3)}(\theta)$ displays a peak around 60°, typical of a HS-like packing (regular tetrahedron). On the other hand, for the covalent-bonded CRN structure, it shows a peak around the tetrahedral bond angle of 109.5°. By contrast, the $g^{(3)}(\theta)$ of *l*-Sn shows a bimodal distribution at bond angles lower than 109.5° (a clear peak centered around 60° and a broad peak centered around 100°).

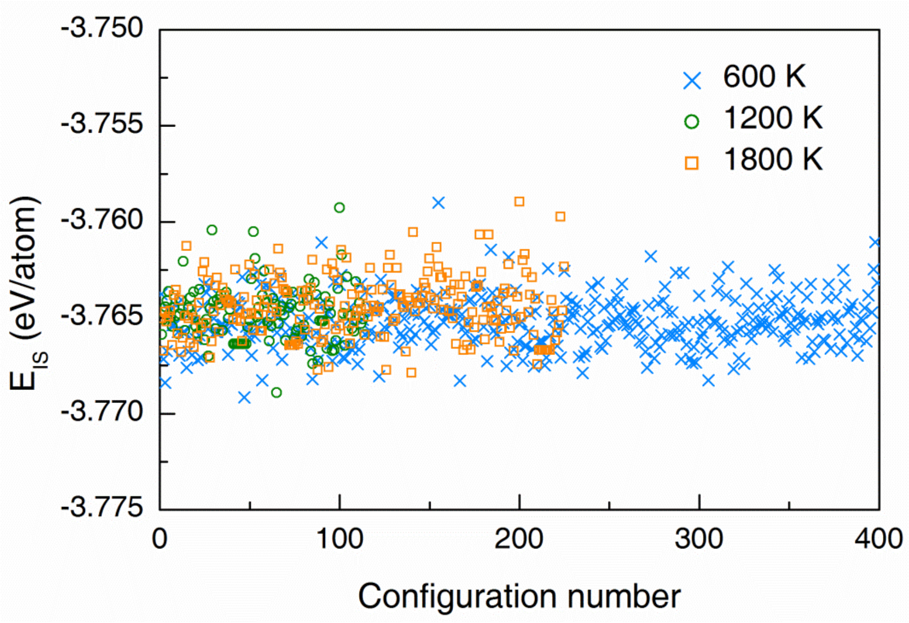

Supplementary Fig. 14. The potential energy of the inherent structures of liquid Sn at different temperatures sampled by isochoric AIMD. The inherent structure energies of *l*-Sn are found to be nearly the same regardless of the liquid temperature, indicating that the liquids have the similar inherent structures, and the structural transition in *l*-Sn is presumably due to vibrational free energy[37].

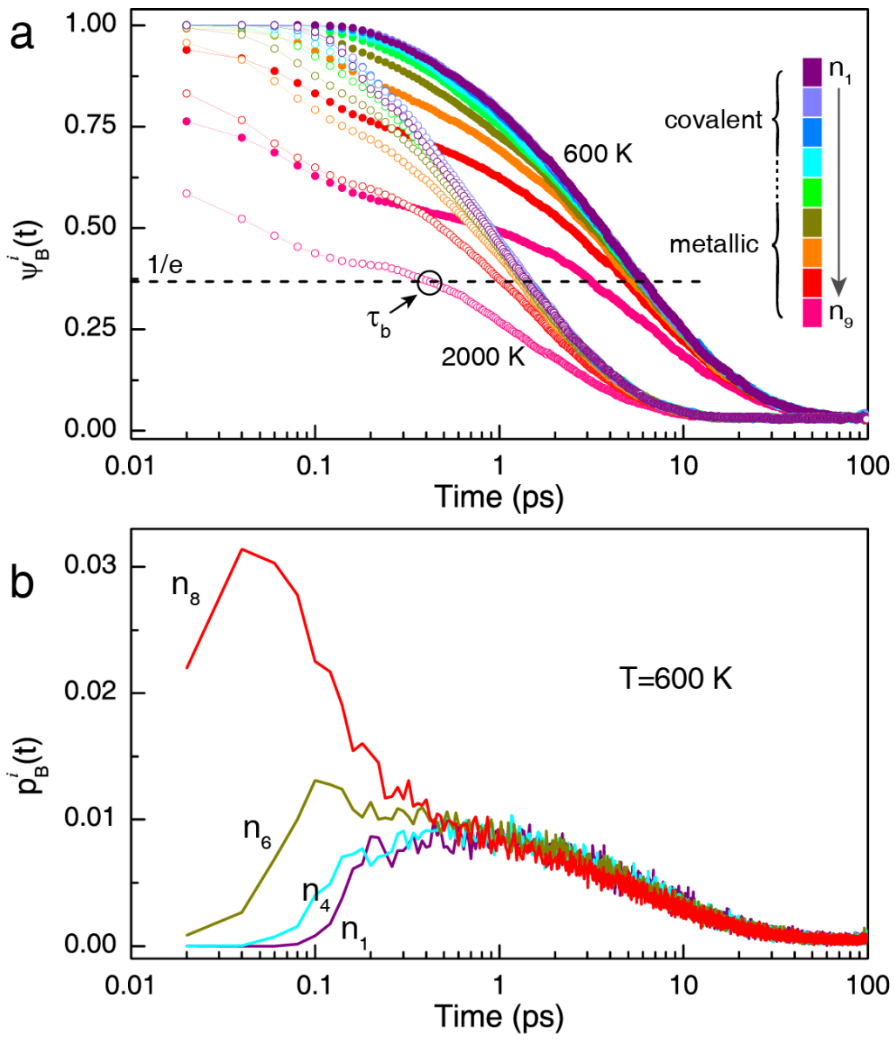

Supplementary Fig. 15. Bond relaxation dynamics revealed by bond-bond autocorrelation function from AIMD. (a) Bond autocorrelation function $\Psi^i_B(t)$ at 600 K (solid circle) and 2000 K (open circle). Bond relaxation time is different for the covalent bonds and the metallic bonds in the liquid. At short relaxation times, the covalent bonds relax slower than the metallic bonds. n_i denotes the ith atomic bond. **(b)** Bond lifetime probability distribution function, $p^i_B(t)$, at 600 K for different types of bonding in *l*-Sn at 600 K. At short times, metallic bonding dissipates faster than the covalent bonding. For longer times, the lifetimes of covalent and metallic bonds are essentially the same, and time-averaged identities of the bonds become indifferentiable.

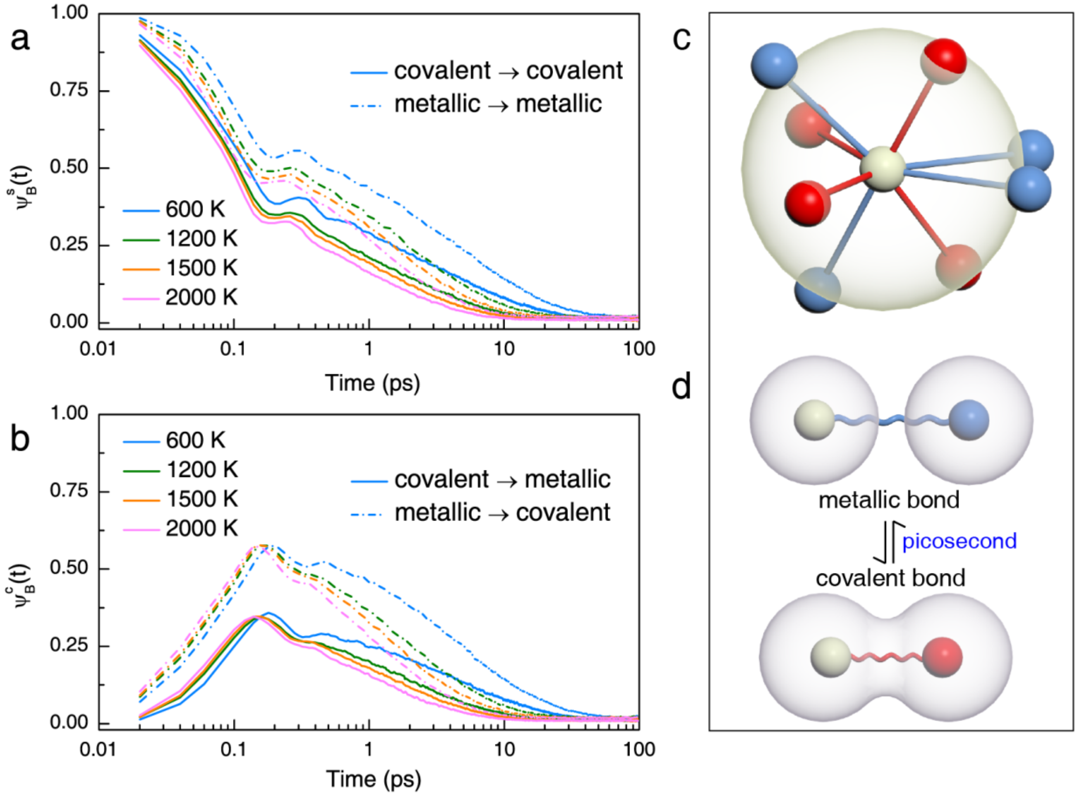

Supplementary Fig. 16. Cross-transition and self-transition between covalent bonds and metallic bonds in liquid Sn. (a) The self-part of the bond correlation function shows the persistence of covalent and metallic bonds in the liquid. **(b)** The cross-part of the bond correlation function shows the mutual transition between the covalent and metallic bonds. **(c)** At a given time *t*, each atom in the liquid is surrounded by atoms with both covalent bonding (red) and metallic bonding (blue). **(d)** Schematic illustration showing the dynamical transition between covalent and metallic bonding. Atomic bonds fluctuate between the metallic and covalent characters within a picosecond timescale, manifesting the fluctuating nature of the atomic bonds in *l*-Sn.

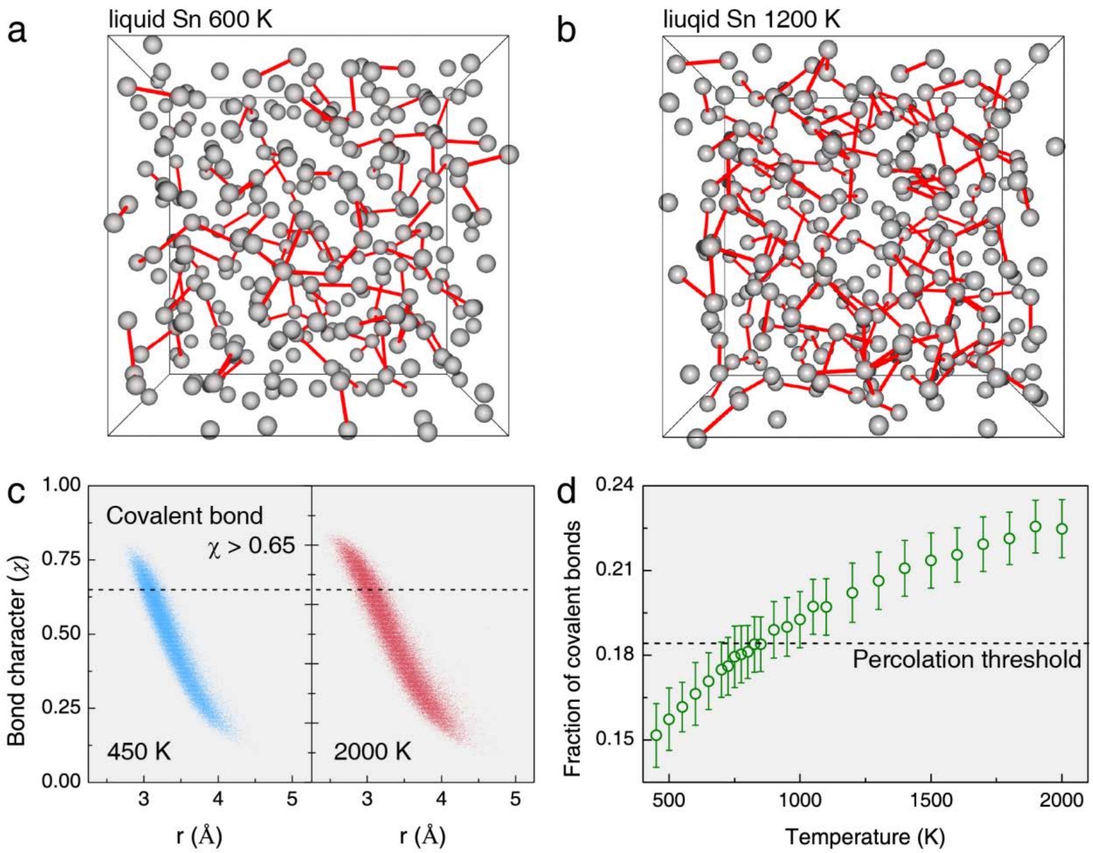

Supplementary Fig. 17. Covalent-bond percolations in liquid Sn. The connectivity of percolating bonds (red lines) in *l*-Sn at **(a)** 600 K and **(b)** 1200 K. **(c)** Bond character (χ) as a function of distance (r) at 450 K and 2000 K, respectively. Strong covalent bonds (connected in red at (a) and (b)) are determined by setting $\chi > 0.65$. Upon increasing temperature, the number of covalent bonds increases. **(d)** Fraction of covalent bonds as a function of temperature. The fraction of covalent bonds of *l*-Sn (Z ≈ 9, $p_c \approx 0.1838$) at T_c is close to the bond-percolation threshold of the bcc lattice (Z = 8, $p_c \approx 0.1803$)[38]. The error bars indicate standard error of the mean. The emergence of the percolating network is responsible for the second-order-like transition (a percolation transition) in *l*-Sn.

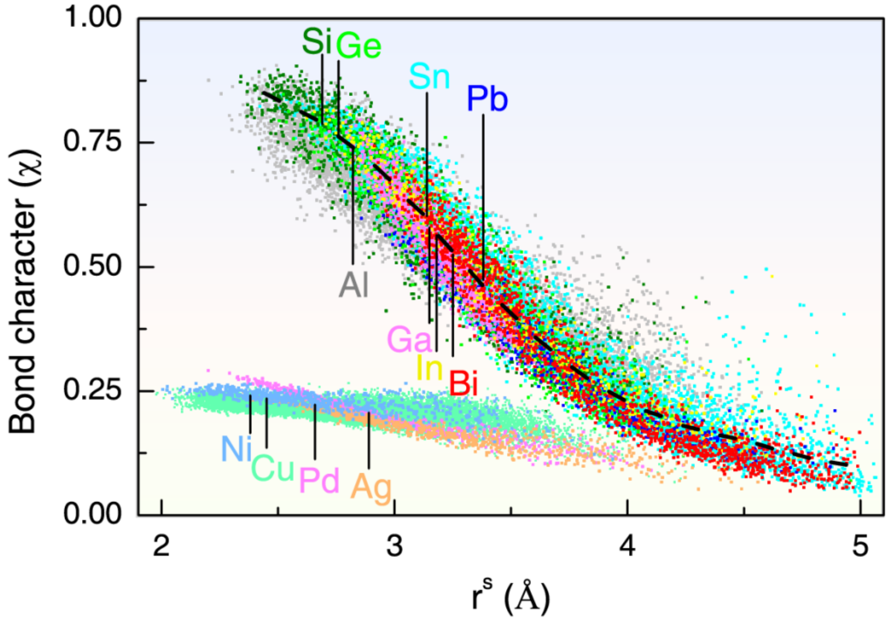

Supplementary Fig. 18. Two types of bonding behavior are identified in pure liquid metals. For normal liquids typified by late transition metals, atomic bonding is predominantly metallic, and the liquids can be described by the HS model. In contrast, (metallic) liquids of polyvalent elements (group III, IV, V elements as indicated in the figure) show dispersive bonding characters. Covalent bonding exists in these liquids at short interatomic distances. For this group of liquid metals, the bonding character as a function of scaled distance r^s falls on a master curve (dashed line), indicating the generality of the varying bonding behavior. As a consequence of the wide spectrum of bonding behavior, the folded-network structure presented in the main text is applicable to this group of liquids. The vertical lines correspond to the peak positions of the pair distribution functions of the liquids (see Supplementary Table 2), indicating different degrees of covalency of the liquids.

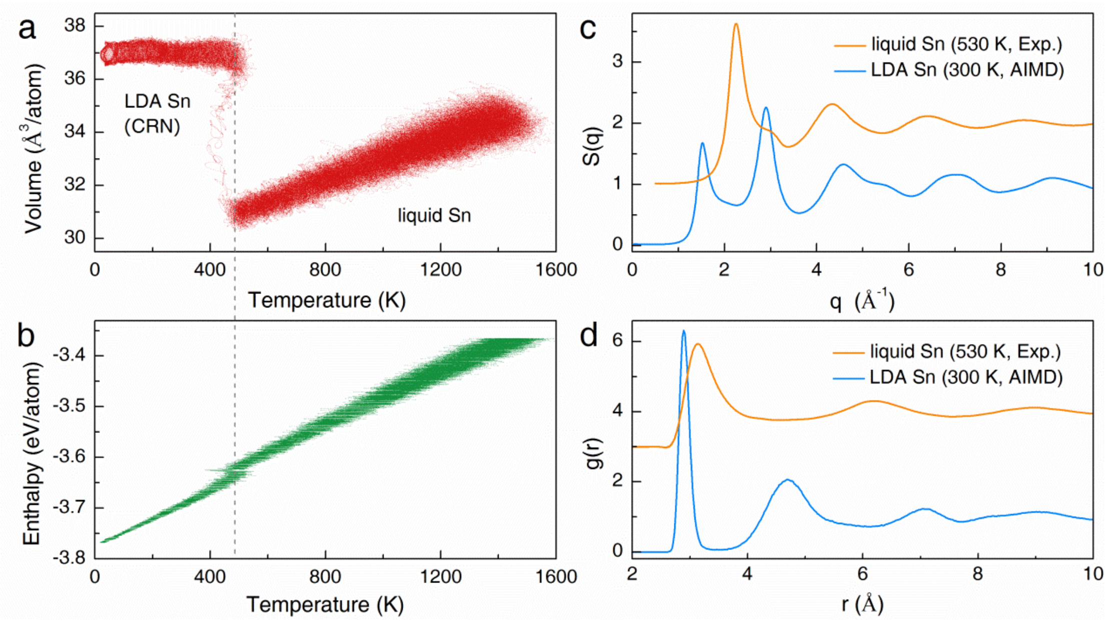

Supplementary Fig. 19. The phase transition between LDA-Sn and liquid Sn. (a) and (b) The volume and enthalpy of CRN and liquid Sn as a function of temperature at 0 GPa from AIMD. The dotted lines show the kinetic spinodal (mechanical instability) of the LDA-Sn upon heating. Upon transition, a volume collapse of ~20% is found. **(c) and (d)** $S(q)$ and $g(r)$ of LDA-Sn employing AIMD and *l*-Sn from the experiment, showing the differences between CRN and the folded network (see Fig. 6 in the main text for atomic configurations). The first-order transition from LDA-Sn to HDL-Sn can be understood in terms of bond-folding in the CRN LDA-Sn that leads toward a folded-network liquid structure at high temperatures.

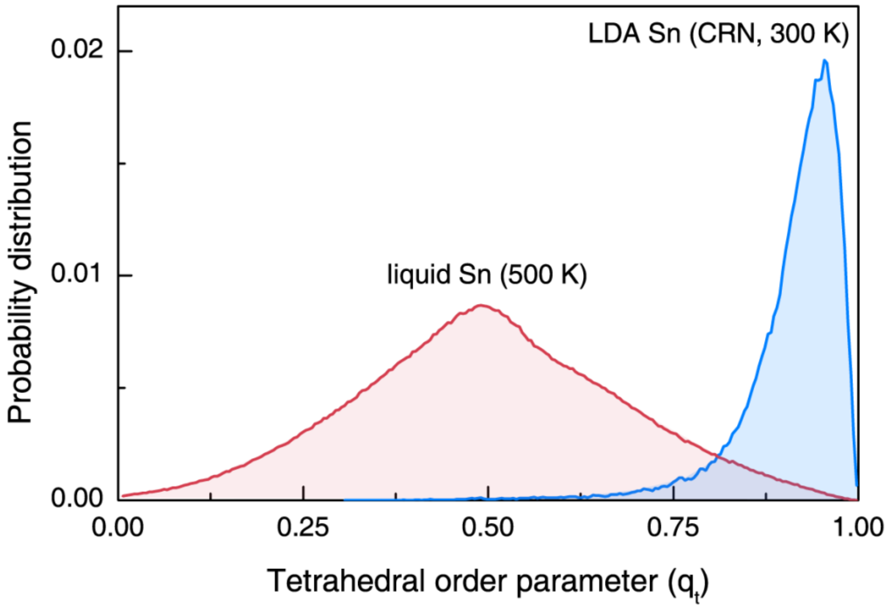

Supplementary Fig. 20. Distributions of tetrahedral order parameter (q_t) for CRN and liquid Sn. $q_t = 1 - \frac{3}{8}\sum(\cos\theta_{ij} + \frac{1}{3})^2$, where θ_{ij} is the bond angle between its four nearest neighbors *i* and *j*, $q_t = 0$ for completely random configurations, $q_t = 1$ for perfectly tetrahedral ordering. For the CRN LDA-Sn structure, q_t is narrowly distributed between 0.75 ~ 1, in agreement with the nearly perfect tetrahedral order of the structure. In contrast, for *l*-Sn, q_t has a much wider distribution, indicating that the tetrahedral arrangement of the shortest bonds is highly distorted, which is consistent with the picture that the bond-angles of *l*-Sn exhibit a large degree of flexibility.

Supplementary Table 1. Ring statistics of n-member rings in the CRN, FN structure and HS models for disordered Sn. An n-membered ring is a ring containing n atoms. The ring statistics indicate the network nature of both structures; but in the FN model, as expected, the ring sizes are smaller (see Fig. 6 in the main text).

n-member rings	CRN (%)	FN (%)	**HS (%)**
n = 4	**20.2**	**30.4**	**0**
n = 5	**25.9**	**29.3**	**0**
n = 6	**26.5**	**23.4**	**0**
n = 7	**22.4**	**15.4**	**0**
n = 8	**5.0**	**1.5**	**0**

Supplementary Table 2. Liquid elements simulated at given volume and temperature (at approximately zero pressure). The radial distance r_0 is scaled to show the generic bonding behavior of the liquids, where the bond characters fall on a master curve (Supplementary Fig. 18). For VASP simulations, we used the PW exchange-correlation functionals together with the PAW treatment. Valance electron configurations used in the AIMD are also provided.

Element	Valance electrons	r_0 (Å)	r_0^s (Å)	V (Å^3/atom)	T (K)
**Si**	$3s^2 3p^2$	2.43	2.69	19.35	1400
**Ge**	$4s^2 4p^2$	2.69	2.76	22.62	1250
**Sn**	$5s^2 5p^2$	3.09	3.14	30.00	2500
**Pb**	$6s^2 6p^2$	3.37	3.38	31.25	1000
**Al**	$3s^2 3p^1$	2.66	2.82	17.23	2200
**Ga**	$4s^2 4p^1$	2.89	3.15	19.42	400
**In**	$5s^2 5p^1$	3.18	3.18	29.41	1000
**Bi**	$6s^2 6p^3$	3.25	3.25	36.54	500
**Ni**	$3d^8 4s^2$	2.38	2.38	12.61	2200
**Cu**	$3d^9 4s^2$	2.45	2.45	12.66	1500
**Pd**	$4d^8 5s^2$	2.66	2.66	17.36	2500
**Ag**	$4d^9 5s^2$	2.89	2.89	21.30	2000

Supplementary References

1 Sheng, H. W. *et al.* Polyamorphism in a metallic glass. *Nat. Mater.* **6**, 192 (2007).
2 Balyuzi, H. H. M. Analytic approximation to incoherently scattered X-ray intensities. *Acta Cryst. A* **31**, 600-602 (1975).
3 Thijsse, B. The accuracy of experimental radial distribution functions for metallic glasses. *J. Appl. Crystallogr.* **17**, 61-76 (1984).
4 Krogh-Moe, J. A method for converting experimental X-ray intensities to an absolute scale. *Acta Crystallogr.* **9**, 951-953 (1956).
5 Norman, N. The Fourier transform method for normalizing intensities. *Acta Crystallogr.* **10**, 370-373 (1957).
6 Kaplow, R., Strong, S. L. & Averbach, B. L. Radial Density Functions for Liquid Mercury and Lead. *Phys. Rev.* **138**, A1336-A1345 (1965).
7 Press, W. H., Teukolsky, S. A., Vetterling, W. T. & Flannery, B. P. *Numerical Recipes 3rd Edition: The Art of Scientific Computing*. (Cambridge University Press, 2007).
8 Sharafat, S. & Ghoniem, N. Summary of Thermo-Physical Properties of Sn, And Compounds of Sn-H, Sn-O, Sn-C, Sn-Li, and Sn-Si And Comparison of Properties of Sn, Sn-Li, Li, and Pb-Li. (M. A. E. Dept, University of California, Los Angles, 2000).
9 Landau, L. D. & Lifshitz, E. M. *Statistical physics (Third Edition), Part 1:* 333 (Pergamon Press, 1980).
10 Angell, C. A. & Zhao, Z. Fluctuations, clusters, and phase transitions in liquids, solutions, and glasses: from metastable water to phase change memory materials. *Faraday Discuss.* **167**, 625-641 (2013).
11 Assael, M. J. *et al.* Reference Data for the Density and Viscosity of Liquid Copper and Liquid Tin. *J. Phys. Chem. Ref. Data* **39**, 033105 (2010).
12 Chandler, D. & Oppenheim, I. Fluctuation Theory and Critical Phenomena. *J. Chem. Phys.* **49**, 2121-2127 (1968).
13 Parrinello, M. & Rahman, A. Crystal Structure and Pair Potentials: A Molecular-Dynamics Study. *Phys.rev.lett* **45**, 1196-1199 (1980).
14 Parrinello, M. & Rahman, A. Polymorphic transitions in single crystals: A new molecular dynamics method. *J. Appl. Phys.* **52**, 7182-7190 (1981).
15 Birch, F. The Effect of Pressure Upon the Elastic Parameters of Isotropic Solids, According to Murnaghan's Theory of Finite Strain. *J. Appl. Phys.* **9**, 279-288 (1938).
16 Buchenauer, C. J., Cardona, M. & Pollak, F. H. Raman Scattering in Gray Tin. *Phys. Rev. B* **3**, 1243-1244 (1971).

17 Kumar, S., Bouzida, D., Swendsen, R. H., Kollman, P. A. & Rosenberg, J. M. The Weighted Histogram Analysis Method for Free-Energy Calculations on Biomolecules. I. The Method. *J. Comput. Chem.* **13**, 1011-1021 (1992).

18 Chandrachud, P., Joshi, K. & Kanhere, D. G. Thermodynamics of carbon-doped Al and Ga clusters: Ab initio molecular dynamics simulations. *Phys. Rev. B* **76**, 235423 (2007).

19 Shell, M. S., Panagiotopoulos, A., Pohorille, A. in *Free Energy Calculations: Theory and Applications in Chemistry and Biology* (ed C. Chipot, Pohorille, A.,) 77-116 (Springer-Verlag, 2007).

20 Hill, T. L. *An Introduction to Statistical Thermodynamics*. (Dover Publications, 1986).

21 Nosé, S. A unified formulation of the constant temperature molecular dynamics methods. *J. Chem. Phys.* **81**, 511-519 (1984).

22 Hansen, J.-P. & McDonald, I. R. *Theory of Simple Liquids (Fourth Edition)*. (Academic Press, 2013).

23 Silvi, B. & Savin, A. Classification of chemical bonds based on topological analysis of electron localization functions. *Nature* **371**, 683 (1994).

24 Becke, A. D. & Edgecombe, K. E. A simple measure of electron localization in atomic and molecular systems. *J. Chem. Phys.* **92**, 5397-5403 (1990).

25 Savin, A. *et al.* Electron Localization in Solid-State Structures of the Elements: the Diamond Structure. *Angew. Chem. Int. Ed. Engl.* **31**, 187-188 (1992).

26 Gibbs, G. V. *et al.* A mapping of the electron localization function for earth materials. *Phys. Chem. Miner.* **32**, 208-221 (2005).

27 Xu, M., Cheng, Y. Q., Sheng, H. W. & Ma, E. Nature of Atomic Bonding and Atomic Structure in the Phase-Change $Ge_2Sb_2Te_5$ Glass. *Phys. Rev. Lett.* **103**, 195502 (2009).

28 Moss, S. C. & Graczyk, J. F. Evidence of Voids Within the As-Deposited Structure of Glassy Silicon. *Phys. Rev. Lett.* **23**, 1167-1171 (1969).

29 Temkin, R. J., Paul, W. & Connell, G. A. N. Amorphous germanium II. Structural properties. *Adv. Phys.* **22**, 581-641 (1973).

30 Wooten, F., Winer, K. & Weaire, D. Computer Generation of Structural Models of Amorphous Si and Ge. *Phys. Rev. Lett.* **54**, 1392-1395 (1985).

31 Buckel, W. & Hilsch, R. Supraleitung und Widerstand von Zinn mit Gitterstörungen. *Zeitschrift für Physik* **131**, 420-442 (1952).

32 Markiewicz, R. S., Shiffman, C. A. & Ho, W. Superconductivity and disorder-driven metal-insulator transition in quench-condensed tin films. *J. Low Temp. Phys.* **71**, 175-191 (1988).

33 Sheng, H. W., Luo, W. K., Alamgir, F. M., Bai, J. M. & Ma, E. Atomic packing and short-to-medium-range order in metallic glasses. *Nature* **439**, 419 (2006).

34 Itami, T. *et al.* Structure of liquid Sn over a wide temperature range from neutron scattering experiments and first-principles molecular dynamics simulation : A comparison to liquid Pb. *Phys. Rev. B* **67**, 480-485 (2003).

35 Umnov, A. G. & Brazhkin, V. V. Study of liquid and solid tin at high temperatures and high pressures. *High Temp. - High Pressures* **25**, 221 (1993).

36 Brazhkin, V. V., Popova, S. V. & Voloshin, R. N. High-pressure transformations in simple melts. *High Pressure Res.* **15**, 267-305 (1997).

37 Stillinger, F. H. A Topographic View of Supercooled Liquids and Glass Formation. *Science* **267**, 1935 (1995).

38 Lorenz, C. D. & Ziff, R. M. Precise determination of the bond percolation thresholds and finite-size scaling corrections for the sc, fcc, and bcc lattices. *Phys. Rev. E* **57**, 230-236 (1998).
